# Toward Application of Liquid Crystalline Elastomer for Smart Robotics: State of the Art and Challenges

**DOI:** 10.3390/polym13111889

**Published:** 2021-06-06

**Authors:** Dandan Sun, Juzhong Zhang, Hongpeng Li, Zhengya Shi, Qi Meng, Shuiren Liu, Jinzhou Chen, Xuying Liu

**Affiliations:** 1 School of Materials Science and Engineering, The Key Laboratory of Material Processing and Mold of Ministry of Education, Henan Key Laboratory of Advanced Nylon Materials and Application, Zhengzhou University, Zhengzhou 450001, China; sundd@gs.zzu.edu.cn (D.S.); szybz@gs.zzu.edu.cn (Z.S.); mengq@gs.zzu.edu.cn (Q.M.); cjz@zzu.edu.cn (J.C.); liuxy@zzu.edu.cn (X.L.); 2 School of Mechanical Engineering, Yangzhou University, Yangzhou 225127, China; lihongpeng@yzu.edu.cn

**Keywords:** liquid crystalline elastomer, soft robots, smart robots, liquid crystal

## Abstract

Liquid crystalline elastomers (LCEs) are lightly crosslinked polymers that combine liquid crystalline order and rubber elasticity. Owing to their unique anisotropic behavior and reversible shape responses to external stimulation (temperature, light, etc.), LCEs have emerged as preferred candidates for actuators, artificial muscles, sensors, smart robots, or other intelligent devices. Herein, we discuss the basic action, control mechanisms, phase transitions, and the structure–property correlation of LCEs; this review provides a comprehensive overview of LCEs for applications in actuators and other smart devices. Furthermore, the synthesis and processing of liquid crystal elastomer are briefly discussed, and the current challenges and future opportunities are prospected. With all recent progress pertaining to material design, sophisticated manipulation, and advanced applications presented, a vision for the application of LCEs in the next generation smart robots or automatic action systems is outlined.

## 1. Introduction

The growing need to automate daily tasks is driving the development of human-friendly robotics. Soft robots are novel devices made of soft and extensible materials, such as fluids, gels, and elastomers, instead of rigid links, joints, and motors [1]. Compared with traditional rigid robots, soft robots are elastically soft and have provided more adaptability to the constrained cooperating environments, particularly in a human–robot integrated workspace. The actuators enabling the generation of mechanical motion are essential for soft robots to provide various kinds of automatic actuation [2]. According to the actuation mechanisms, soft robots can be divided into two categories: (1) Continuum robots, depending on traditional motors and transmission mode (such as gears and ball screws). (2) Soft robots based on the actuation of soft smart materials. Among them, soft robots based on smart materials can directly convert some external stimuli into mechanical work, providing more application prospects in cutting-edge fields, such as micro-electromechanical systems, microrobots, sensors, etc. [3]. Commonly used soft smart materials for soft robots include shape memory polymers (SMPs) [4,5,6,7], electroactive polymers (EAPs) [8], hydrogels [9], and liquid crystalline elastomers (LCEs) [10,11,12].

LCEs are lightly crosslinked polymer networks with incorporated rigid and anisotropic mesogens units. As representative anisotropic materials, LCEs can achieve contactless motility under external stimuli through programmed molecular orientations [13]. The mesogens in LCEs can exhibit spontaneous orientational ordering, which is similar to that observed in ordinary low-molar-mass liquid crystals (LCs). Therefore, the LCE networks will contract along the LC direction and expand in the vertical direction with the order–disorder transition of mesogens, resulting in the macroscopic shape changes of the bulk LCEs [14,15]. Similar to small molecule liquid crystals, the phase transition of LCEs can be achieved by environmental stimuli, such as temperature [16,17], humidity [18,19], organic solvent [20,21], electric [22,23,24,25,26,27], and ion [28] or remote stimuli, such as light [29], magnetic field [30,31,32], etc. Furthermore, the shape change of LCEs is fully reversible since their phase transition is dictated by thermodynamic equilibrium [33]. Fully reversible shape-changing, good reliability, suitable elastic modulus, and large actuated strain make LCEs an ideal candidate for actuators, sensors, and smart robots.

**Figure 1:**
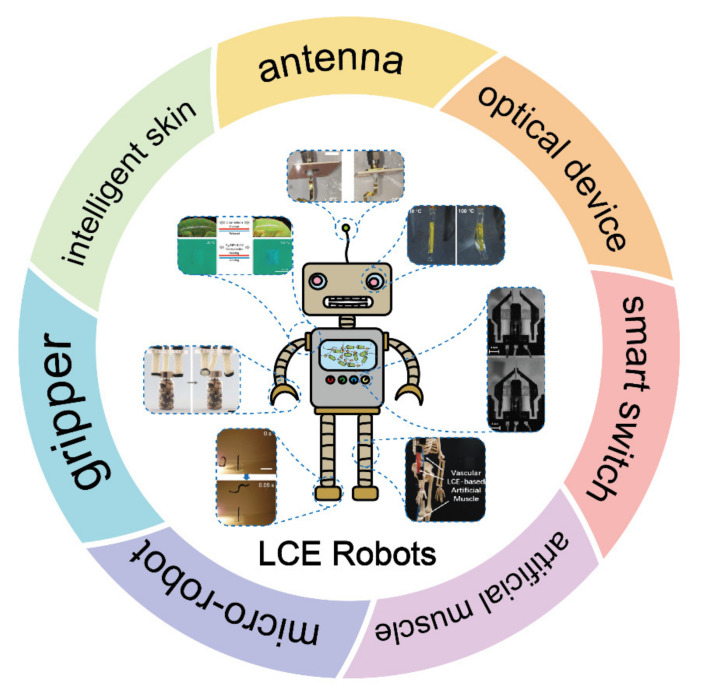
Applications of liquid crystalline elastomers in optical device [11] (Reproduced with permission from López-Valdeolivas, M.; Liu, D.; Broer, D.J.; Sánchez-Somolinos, C., Macromol. Rapid Commun.; Copyright 2018 John Wiley and Sons.), smart switches [34] (Reproduced with permission from Sánchez-Ferrer, A.; Fischl, T.; Stubenrauch, M.; Wurmus, H.; Hoffmann, M.; Finkelmann, H, Macromol. Chem. Phys.; Copyright 2009 John Wiley and Sons.), artificial muscles [35] (Reproduced with permission from He, Q.; Wang, Z.; Song, Z.; Cai, S., Adv. Mater. Technol.; Copyright 2019 John Wiley and Sons.), micro-robots [36] (Reproduced with permission from Ahn, C.; Liang, X.; Cai, S., Adv. Mater. Technol.; Copyright 2019 John Wiley and Sons.), soft grippers [37] (Reproduced with permission from He, Q.; Wang, Z.; Wang, Y.; Minori, A.; Tolley, M.T.; Cai, S., Science Advances; Copyright 2019 American Association for the Advancement of Science.), intelligent skins [38] (Adapted from Ref. [38] with permission from the Centre National de la Recherche Scientifique (CNRS) and The Royal Society of Chemistry.), and antennas [39] (Adapted with permission from Kim, H.; Gibson, J.; Maeng, J.; Saed, M.O.; Pimentel, K.; Rihani, R.T.; Pancrazio, J.J.; Georgakopoulos, S.V.; Ware, T.H. Responsive, 3D Electronics Enabled by Liquid Crystal Elastomer Substrates. ACS Appl. Mater. Interfaces 2019, 11, 19506–19513, doi:10.1021/acsami.9b04189. Copyright (2019) American Chemical Society).

In this review, the recent advances in LCE-based smart automatic devices are overviewed, including optical devices, smart switches, artificial muscles, micro-robots, soft grippers, intelligent skins, and antennas (Figure 1). Additionally, their unique advantages, good performance, and potential for future applications of smart robots are highlighted. Furthermore, the synthesis methodologies, structures, and alignment strategies of LCEs are briefly discussed in Section 2. By discussing the existing challenges and future perspectives of LCEs in smart devices, this review is useful to provide a comprehensive understanding of the current status of LCEs to promote the application of LCEs in smart robots and other locally controllable integrated automatic system.

## 2. Structure and Preparation of LCEs

**Figure 2:**
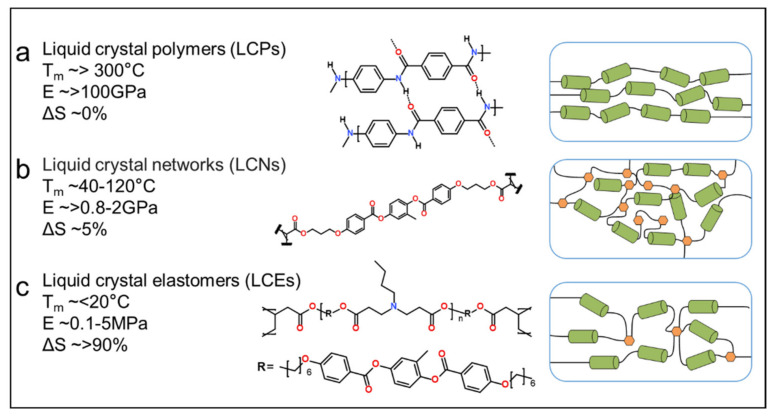
Notional properties and molecular configurations of LCPs (**a**), LCNs (**b**), and LCEs (**c**).

At present, three kinds of polymers with liquid crystal (LC) properties have been explored, namely, liquid crystalline polymer (LCP), liquid crystalline polymer network (LCN), and LCE. The differences in the structure and chemical properties of these materials are shown in Figure 2. Typically, LCPs are uncrosslinked polymers in which macromolecules can be organized into liquid crystalline phases through molecular conformation and intramolecular interactions [40]. LCN retains some of the properties of LCP but, significantly, contains a moderately to highly crosslinked network. LCE exhibits a similar crosslinked network structure compared with LCN, but the overall crosslinking density is relatively low [40]. The structural differences between the above materials result in many differences in their properties. For instance, LCP is a good choice for engineering plastics because of its good strength and low dielectric constant, caused by its significant stable higher-order structure (shows almost no change of order under external stimulus). LCNs can produce mechanical actuation in response to external stimuli as their order (described by the order parameter, S) can be decreased by as much as 5% at this point. Unlike LCPs or LCNs, LCEs can exhibit large changes in order (>90%) when subject to a stimulus, thus, providing better flexibility and greater reversible deformation capability [40].

Generally, to enable the anisotropic properties of LCEs, mesogenic moieties must be incorporated into the polymer network, either in chain backbone or as side groups. Therefore, LCEs can be divided into main-chain and side-chain LCEs according to the location of mesogens. In main-chain LCEs, mesogens are polymerized with other monomers to construct the polymer backbone, while for the side-chain LCEs, mesogens are attached as side chains of the polymer by spacers. LCEs containing rod-shaped mesogens can also be classified into two categories according to their geometric positions: the end-on LCEs and the side-on LCEs. For end-on LCEs, rod-shaped mesogens are connected with the chain of the polymer along the long axis, while in the side LCE, the rod-shaped LC unit is connected along the short axis. In addition, the phase regime and the polymorphism of LC in LCEs can be specifically affected by these above geometries. For example, side-on side-chain LCEs tend towards the formation of nematic phases, while the end-on side-chains are more likely to form a smectic phase.

### 2.1. Synthesis and Alignment of LCEs

Depending on the precursors, the strategies for the synthesis of LCEs can be divided into two categories: (1) the pathways employing at least one polymeric precursor or (2) those exclusively using monomers as precursors. For the first category, poly (hydrosiloxane) polymers or liquid crystalline polymers are usually used as precursors (Table 1(a,b)). Among them, the synthesis based on organosilicon chemistry is one of the most commonly used methods, by which the crosslinking density of the polymer networks can be easily controlled by changing the function of the precursor [41,42]. However, for these methods, the low-molecular-mass substances still remained in the elastomer network after the reaction, resulting in phase transfer or separation of the obtained LCEs. Meanwhile, this method is unsuitable for the preparation of main-chain LCEs due to the presence of reactive sites in the poly(hydrosiloxane) precursors. For the strategies using liquid crystal polymers or oligomers as precursors, both side-chain and main-chain LCEs can be achieved [43]. It should be noted that when the LC prepolymer containing cross-linkable group is selected, the reaction can be carried out without solvents [43]. Another category of strategies is directly synthesizing LCE with low-molar-mass monomers. For these strategies, polymerizable LC monomers, free radical initiators, and crosslinking agents were simply mixed and then subjected to be polymerized under ultraviolet or heating conditions. In addition, the molecular weight of the LC monomers used in these reactions is relatively low, providing more convenience for the processing of LCEs. One of the common reactions for this strategy is the “click” reaction (Table 1(d–f)), which has been introduced into the synthesis of LCEs in recent years. In particular, through the utilization of thiol-ene (Table 1(d)) and thiol-acrylate (Table 1(e)) click chemistry, researchers are able to more easily prepare optimized and functionalized LCEs by changing the spacer. The introduction of this synthesis method has allowed researchers from other related disciplines to easily synthesize LCEs through commercially available chemicals.

Dynamic covalent bonds are a class of covalent bonds that can undergo exchange reactions with specific external stimuli [44,45]. The exchange reaction leads to network rearrangement of the materials, providing new functions such as self-healing, welding, reprocessing, and some specific stimulus responsiveness for polymeric material. To address the adaptability and recyclability of LCEs, a number of novel chemical transformations, such as boronic ester exchange reaction [46], disulfide metathesis reaction [47,48,49], reversible addition fragmentation chain-transfer reactions [50,51,52], and siloxane exchange reactions [53,54], have been introduced into LCEs. The general reaction scheme and conditions of dynamic chemistries are given in Table 1(g–o). The ability of breaking, reforming, or exchanging crosslinks or chains in a dynamic network means that, in theory, the network architecture and drive capabilities of LCEs can be redefined permanently [55]. Therefore, dynamic chemistry offers an exciting additional way to process and reprocess LCE materials.

**Table 1:** Chemical reactions for liquid crystalline elastomers.

Types of Chemical Reactions		General Reaction Scheme and Conditions ^a^	Ref.
Hydrosilylation Reaction		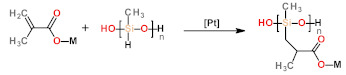 a 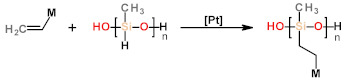 b 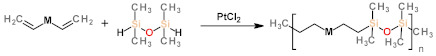 c	[42,56,57,58,59,60]
click reaction	Photo-initiated thiol-ene Michael addition	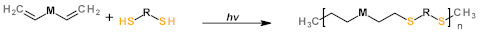 d	[61,62,63]
	Nucleophile-catalyzed thiol-acrylate Michael addition	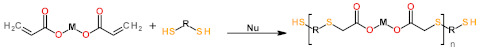 e	[64,65,66]
	Nucleophile-catalyzed aza-Michael addition	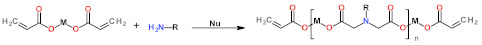 f	[67,68,69]
Associative	Transesterification	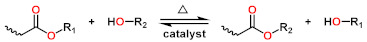 g	[59,70,71]
	Siloxane exchange reaction	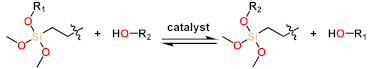 h	[53,54]
	Transcarbamoylation	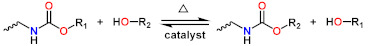 i	[72]
	Boronic ester exchange reaction	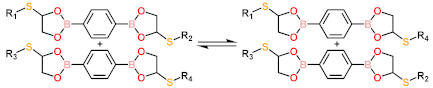 j	[46]
Dissociative and associative	Reversible addition fragmentation chain-transfer reaction	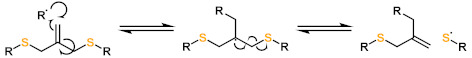 k	[50,51,52]
Dissociative	Disulfide metathesis reaction	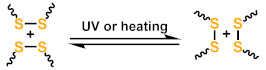 l	[47,48,49]
	Furan-maleimide Diels–Alder reaction	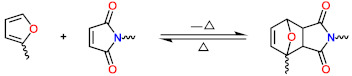 m	[73,74]
	Photodimerization of cinnamate	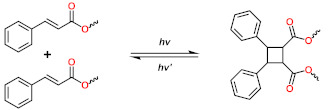 n	[75,76,77]
	Photodimerization of anthracene	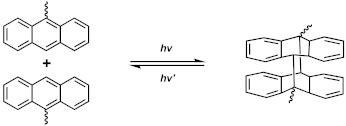 o	[78]

a. M stands for mesogen.

When LCEs are synthesized without additional treatment, the so-called polydomain elastomers with similar macroscopic isotropy to polycrystalline materials will be obtained. Although polydomain LCEs exhibit numerous fascinating properties, it is difficult for them to achieve reversible shape change without external loading, which is a prerequisite for actuating performance. Therefore, the main aspect towards the application of LCEs as actuators is the orientation of mesocrystalline to form monodomain LCEs. To date, several methods have been developed to enable the fabrication of monodomain LCEs. One of the most widely used alignment strategies is mechanical orientation, which was initially pursued by Vorländer and Finkelmann, known as the two-step crosslinking process for polysiloxanebased polymers. This common method is initiated from the synthesis of a weakly crosslinked liquid crystalline polymer, and then, the stress is applied to uniaxially stretch the samples, resulting in the expansion of polymer chains, contrary to entropy. In this process, the mesogens in the polymer network can be macroscopically aligned before the precursor is completely crosslinked, after which the conformation of the main chain is locked by a second-stage crosslinking reaction to form highly aligned LCEs.

Some external fields, such as magnetic and electric fields, can also be applied to align LCEs. For example, complex 3D-patterned magnetic fields have been successfully employed to create arrays of microstructures with area-specific molecular orientations [79]. This method is based on the utilization of a magnetic field, by which a magnetic-field-defined uniaxial orientation of the LC director within microstructures of any simple or complex 3D shape can be programmed. After that, the following photopolymerization is conducted to preserve the molecular configuration [80]. Photo-alignment by linearly polarized light is considered a suitable method for obtaining thinner monodomain LCE due to the limited penetration of the polarized light. The particular advantage of this approach is that the spatially complex patterning of surfaces, such as origami-like actuators [21], checkerboards [81], and artificial iris [82], can be achieved by photomasks. In addition, this strategy can also be used to realize voxel-by-voxel resolution molecular programming in LCEs [21], and 3D helix engineered photonic materials can be obtained when the mesogens are chiral nematic LCs [83].

Aligning surfaces are also widely used to induce the uniform orientation of mesogens in thinner materials. In order to establish the orientation of the director in a single plane, some alignment layers, such as rubbed polyimide [84], polyvinyl alcohol [85], or aligned CNT sheets, should first be manufactured. When smectic liquid crystalline polymers or liquid crystal monomer mixtures are spread to alignment layers, the mesogens adopt a homeotropic alignment with respect to the film’s surface driven by the trend of surface energy minimization. In a recent study, Kim et al. [86] used highly aligned CNT sheets as an alignment layer for the LCEs. They found that highly oriented CNT sheets can simultaneously guide the alignment of LC monomers and oligomers.

### 2.2. Novel Molding Methods of LCEs

For many applications of LCEs, their functionality may be greatly restricted if they fail to be processed into appropriate sophisticated and complex geometries or specific microstructures. Therefore, processing of LCEs is a necessary step for their ultimate use. At present, many strategies have been established to realize the various structure manufacturing of LCEs, such as injection molding [87], folding [88], soft lithography [89], microfluidic [90], and several new processing methods, such as 3D printing [25,91,92].

**Figure 3:**
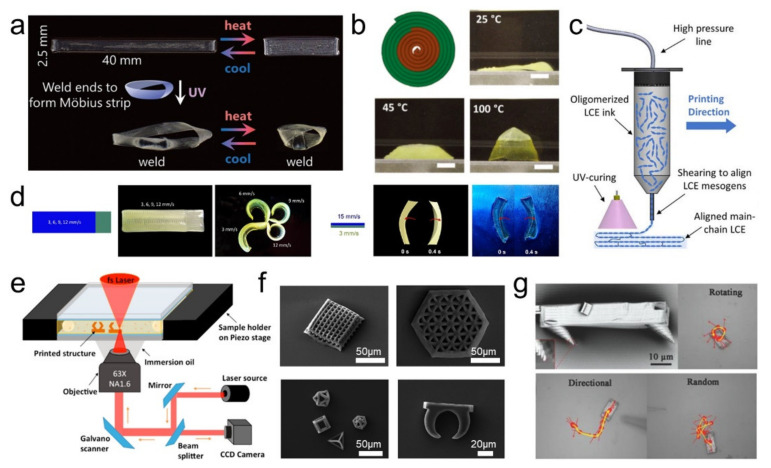
(**a**) A printed strip exhibits reversible linear actuation until the ends are welded together at 60 °C via dynamic bond exchange to form a reversibly actuating Möbius strip [91] (Reproduced with permission from Davidson, E.C.; Kotikian, A.; Li, S.; Aizenberg, J.; Lewis, J.A., Adv. Mater.; Copyright 2020 John Wiley and Sons.), (**b**) Printing schematic and photographs of a disk printed with two different actuation temperatures of LCE materials in a +1 defect print pattern [92] (Reproduced with permission from Saed, M.O.; Ambulo, C.P.; Kim, H.; De, R.; Raval, V.; Searles, K.; Siddiqui, D.A.; Cue, J.M.O.; Stefan, M.C.; Shankar, M.R.; et al., Adv. Funct. Mater.; Copyright 2019 John Wiley and Sons.), (**c**) Schematic of LCE oligomer ink DIW 3D printing [25] (Reproduced with permission from Roach, D.J.; Kuang, X.; Yuan, C.; Chen, K.; Qi, H.J., Smart Mater. Struct.; ©2018 IOP Publishing. Reproduced with permission. All rights reserved.), (**d**) Schematics and photographs of locally programmed popping-up and oscillating behaviors of LCE actuators [101] (Adapted with permission from Ren, L.; Li, B.; He, Y.; Song, Z.; Zhou, X.; Liu, Q.; Ren, L. Programming Shape-Morphing Behavior of Liquid Crystal Elastomers via Parameter-Encoded 4D Printing. ACS Appl. Mater. Interfaces 2020, 11. Copyright (2020) American Chemical Society.), (e) Schematic of femtosecond DLW of 3D microstructures in the cell via two-photon polymerization [102], (**f**) SEM images of the LCE 3D microstructures by using DLW [102] (Adapted with permission from Chen, L.; Dong, Y.; Tang, C.-Y.; Zhong, L.; Law, W.-C.; Tsui, G.C.P.; Yang, Y.; Xie, X. Development of Direct-Laser-Printable Light-Powered Nanocomposites. ACS Appl. Mater. Interfaces 2019, 11, 19541–19553, doi:10.1021/acsami.9b05871. Copyright (2019) American Chemical Society.), (**g**) Photographs of the microscopic artificial walker [103] (Reproduced with permission from Zeng, H.; Wasylczyk, P.; Parmeggiani, C.; Martella, D.; Burresi, M.; Wiersma, D.S., Adv. Mater.; Copyright 2015 John Wiley and Sons).

Three-dimensional (3D) printing is perceived as a promising technology to create sophisticated 3D architectures for a wide range of custom-made materials, including metal, ceramics, as well as smart polymers, such as shape memory polymers, LCEs, etc. [93,94]. 3D printing technology has undergone significant development in recent years, and researchers have fabricated complex shapes that were impossible to achieve with traditional methods [95]. Davidson et al. [91] designed an LCE ink with dynamic bonds for 3D printing. The reconfigurable LCE ink can be locally programmed during the printing process and can be subsequently reconfigured by UV light (Figure 3a). Traugutt, Luo, and their coworkers developed a digital-light-processing (DLP)-printable LC oligomer that allows them to print centimeter-scale isotropic and anisotropic digital lattice devices with high resolution(10 μm) [96,97]. Compared to commercial materials, lattice devices printed from this material have shown far greater levels of strain-rate dependency and load curve hysteresis.

However, the full potential of LCEs cannot be realized by simply manufacturing objects with complex shapes, so 4D printing, a method that enables the programming of LCEs during the 3D printing process, has recently emerged. Time is usually introduced as an additional fourth dimension in 4D printing. When 4D printed structures are exposed to appropriate stimulus conditions, they are able to achieve shape changes in a predetermined manner [98]. Recently, 4D printing was introduced into LCEs in 2017 almost simultaneously by the groups of Ware [99] and Sanchez-Somolinos [11] that allows LCEs to achieve more complex and programmable shape changes. Lu et al. [73] designed a novel 4D-printable azobenzene-functionalized LCE material that combined supramolecular crosslinks and dynamic covalent crosslinks (Diels-Alder). After UV irradiation, the programmable shape changes of the materials are stable, which arises from a decoupling of the isomerization. By utilizing these features, the authors have fabricated a reprogrammable Braille-like actuator. Coupling the conductive properties and deformability of liquid metal with the reversible deformation of LCEs can be used to fabricate multifunctional electrothermal soft actuators. Ambulo et al. [100] developed a process to fabricate 4D-printable LCEs embedded with liquid metal. The liquid metal provides the LCE composites with electro- and photo-thermal actuation capabilities and is capable of producing 12% deformation (under 1.6 V DC) or 150° bending (under 730 nm NIR light irradiation), respectively. Ceamanos et al. [69] designed a 4D printing method of azobenzene-containing LCEs. The deformation or force of the printed objects can be adjusted by controlled light intensities, which is suit for push or pull purposes in complex systems such as soft robots. Saed et al. [92] employed the thiol-ene “click” reaction to synthesize LCEs, which can be 4D printed into complex shapes. With 4D printing, the molecular orientational order of LC inks can be locally programmed to order along the direction of the printed ink paths, thus, the multiple and reversible shape changes of the 4D printed actuator can be realized (Figure 3b). Roach et al. [25] developed an LCE oligomer ink with shear-thinning behavior and favorable viscosity that is suitable for DIW-3D printing (Figure 3c). The ink exhibits a low nematic to the isotropic transition temperature, allowing LCEs to be printed at room temperature with a maximum actuation temperature of 75 °C. In addition, novel structures, including a box, a soft robotic gripper, and a hand with five reversibly actuating fingers, have been successfully fabricated and demonstrated by printing, showing the potential of using LCE for 4D printed active structure applications. Zhang et al. [77] prepared a single-component LCE ink for DIW printing and then used it to fabricate LCE actuators. The actuator can perform reversible bending deformation due to the orientated gradient state, which is caused by the temperature difference between the two sides of the printed samples. Ren et al. [101] put forward the concept of parametric encoding 4D printing. By adjusting the printing parameters during printing, the shape deformation behavior of a specific location can be achieved in a single material (Figure 3d). Furthermore, by modulation of the printing speed in specific regions, they implemented local programming for pop-up, self-assembling, and oscillating behaviors.

The construction of the microstructure can give the material unique properties, such as structural color [38] and superhydrophobic properties [104,105]. Some methods for structure manufacturing have been used for constructing LCEs with specific microstructures. Chen et al. [102] fabricated a series of NIR-driven micro-structured LCEs by using two-photon polymerization lithography (Figure 3e,f). Thanks to the photothermal effect of AuNRs, it can achieve rapidly reversible shape deformation via NIR laser irradiation. Zeng et al. [103] adopted direct laser writing (DLW) to fabricate a light-fueled microscopic artificial walker (Figure 3g) that is smaller than any known terrestrial organism and can perform several autonomous movements on different surfaces.

## 3. Application of LCEs for Soft Robots

Miniaturization is becoming a prominent trend for actuators, robots, and many other smart devices [106]. These small-scale structures and devices are difficult to manufacture with rigid materials and traditional methods. Therefore, soft materials with better flexibility and biocompatibility seem to be promising candidates. LCEs are lightly crosslinked anisotropic polymer networks with attributes of both elastomers and LCs. A notable feature of these materials is that their thermotropic order–disorder transition can induce large strain and reversible shape changes. In addition, some functional fillers can also be introduced into LCE to enable their multiresponse to electrical, light, or other stimuli. Thanks to their salient functionality and flexibility, LCEs have emerged as a new smart actuating material for building novel spontaneous systems and smart robots. By the motion modes of bending, twisting, curling, folding, elongation, and shortening, various actuators and functional soft robots, such as soft grippers, sensors, antennas, and optical devices, etc., have been developed recently.

### 3.1. Actuators Based on LCEs

**Figure 4:**
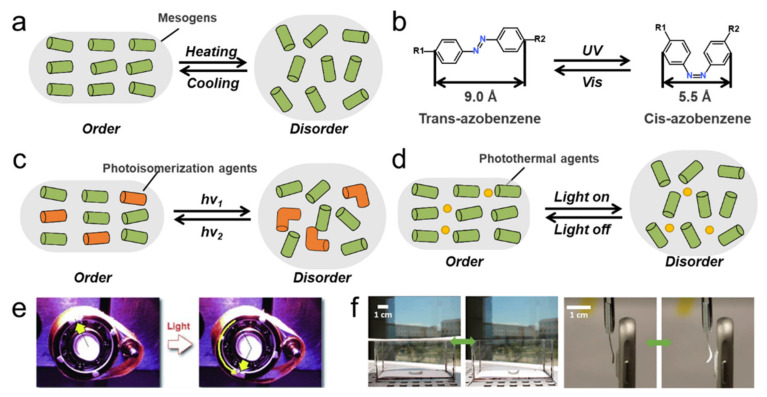
(**a**) Schematic representation of thermal-induced order-disorder phase transition in LCEs. (**b**) Reversible trans-cis photoisomerization of azobenzenes. (**c**) Schematic representation of photo-induced order-disorder phase transition in LCEs. (**d**) Schematic representation of photothermal-induced order-disorder phase transition in LCEs. (**e**) A light-driven plastic motor with the azobenzene-containing LCE laminated film [112] (Reproduced with permission from Yamada, M.; Kondo, M.; Mamiya, J.; Yu, Y.; Kinoshita, M.; Barrett, C.J.; Ikeda, T., Angewandte Chemie International Edition; Copyright 2008 John Wiley and Sons.) (**f**) Photothermal bending actuation triggered by sunlight (100 mW cm^−2^) and the light emitting diode on a mobile phone (150 mW cm^−2^) [86] (Reproduced with permission from Kim, H.; Lee, J.A.; Ambulo, C.P.; Lee, H.B.; Kim, S.H.; Naik, V.V.; Haines, C.S.; Aliev, A.E.; Ovalle-Robles, R.; Baughman, R.H.; et al., Adv. Funct. Mater.; Copyright 2019 John Wiley and Sons).

Thermal-mechanical response is one of the most common characteristics of LCEs. Similar to low-molar-mass LCs, loosely crosslinked LCEs often exhibit thermotropic properties. Upon their clear point, the phase transition from the LC phase to the isotropic state will trigger a large shrinkage strain parallel to the director, resulting in a shape change in LCEs (Figure 4a). In recent years, a series of studies on the thermodynamic properties of LCEs have been carried out. Saed et al. [107] studied the effect of the type and concentration of crosslinker on the thermomechanical properties of main-chain LCEs. The results show that the isotropic transition temperature (T_i_) of LCEs can be influenced by the functionality of the crosslinker and their actuation performance can be tailored by controlling the amount of crosslinker and applied stress. Another work from them [108] investigated the influence of spacer length on the properties of main-chain LCEs. They confirmed that longer spacers could drive nano-scale segregation in the polymeric network to get smectic phases, while nematic phases will be obtained by employing shorter spacers. Compared to nematic networks, smectic networks show larger magnitudes of actuation. Traugutt et al. [16] compared the influence of liquid-crystal orientation on the thermo-mechanical properties of the main chain LCEs, they found that the actuation performance, work capacity, dissipation, and elastic modulus of the LCEs are all dependent on the alignment state of the mesogenic unit during synthesis.

Actuation by light is a promising strategy for the action of LCEs due to its distinct advantages of wireless control, abundant light sources, and wavelength selectivity [109]. There are many methods for realizing the photoresponse of LCEs. A more common method is introducing photoresponsive chromophore (like azobenzenes) into the LCE matrix to realize the conversion of light energy into mechanical energy by changing the structure of the chromophore upon light stimulation. The reversible cis-trans isomerization of azobenzene can be described as a geometric isomerization, which is always accompanied by a significant change in molecular length (Figure 4b) [110] Thus, when azobenzene chromophores are added to the backbone of the crosslinked LC network, reversible shrinkage and expansion of the monolithic material can be obtained by changing the wavelength of the light source (Figure 4c). Finkelmann et al. [111] first reported the photo-induced shrinkage behavior of monodomain nematic LCEs with polysiloxane as the main chain and azophenyl groups as chromophores. Later, Yamada et al. [112] reported an azobenzene-containing LCE laminated film and further use it to develop a micro-motor that could run under light stimulation (Figure 4e). The light-driven motor is composed of pulleys, axles, and a belt connecting both ends of the LCE laminated film. When the upper right and upper left sides of the belts are irradiated with UV and visible light, respectively, contraction stress is generated on both the right pulley and the left pulley to make it rotate counterclockwise; thus, the micromotor can rotate continuously. In order to study the deformation process of photochemical LCEs, some mathematical models have been used to predict the mechanical response under specified target loading conditions [113,114,115]. Recently, Bai and Bhattacharya [116] theoretically explored the photomechanical coupling in a photosensitive LCE under light irradiation and mechanical stress. They demonstrated the induced large deformation and the formation of stripe domains during the transition between mechano-alignment and photo-alignment. However, such light-stimulated responsive LCEs based on photochemical reactions have some drawbacks; for example, the strong light absorption of azo-based chromophore groups restricts the light transmission depth.

Thermally actuated LCEs can be easily converted into light-driven actuators by using photothermal effects (Figure 4d). Many photothermal fillers, such as carbon nanotubes (CNTs) [70,117,118,119,120,121,122], graphene oxides [123], dyes [29,122,124,125,126,127,128,129], gold nanorods [130,131], nanoparticles [132], conjugated polymers [133], and polydopamine [134,135], have been successfully introduced into LCEs to enable their light responsiveness. Kim et al. [86] presented a strategy for preparing programmable, multiresponse LCE/CNT composites that can respond to both optical (Figure 4f) and electrical stimuli by introducing CNT functional fillers into LCE matrix. In the nanocomposites, CNT fillers not only play the roles of traditional mechanical reinforcement, thermal conductivity, and conductivity enhancement but also act as an alignment layer of LCEs. By controlling the orientation, location, and quantity of CNT layers in LCE/CNT composites, patterned actuators that can present rapid, reversible, programmed, and wireless multiresponse to visible light or electrical current are successfully constructed [86].

Recently, bilayer-structured actuators based on LCE stimuli-responsive layers have attracted much attention. Kohlmeyer et al. [122] assembled an active IR-active fillers/LCE composite layer with a passive silicone resin into bilayer, wavelength-selective, IR light-driven hinges. The LCE composite/silicone bilayer hinges not only exhibit fast and reversible bending due to the bulk N-I LCE phase transition but also exhibit infrared wavelength selectivity by using NIR dye as fillers. Xing et al. [136] prepared a novel thermally responsive bilayer actuator by filling LCE monomers into a three-dimensional SiO_2_ PC template. The macroscopic shape and the spectrum of the composite film can be simultaneously controlled by thermal switching of molecule orientation in LCEs. As the temperature increases, the SiO_2_ PC/LCE composite film exhibits significant bending deformation, and its photonic band gap shifts to a shorter wavelength at the same time, which will be of interest in designing optical actuator systems for environment-temperature detection. Reciprocal oscillatory motion is important for micromachines, such as mechanical watches, micro-robots, and ventricular assist devices. Vantomme et al. [137] reported joint LCN actuators that can oscillate similar to the synchronized motion of pendula and metronomes. They found that two joint LCN oscillators driven by light can communicate and synchronize their oscillations together, similar to results Huygens observed with pendulum clocks. Since this synchronization is sensitive to changes in the stiffness and damping of the joint, it could be used to sense the material’s mechanical properties.

### 3.2. LCEs as Grippers

In nature and engineering applications, an actuator enabling gripping often can be found, such as an elephant’s trunk, octopus’s arm, and starfish’s tube foot. Concentric tubular surgical robots [138,139] and endoscopes [140,141] are typical examples in biomedical engineering that can realize multimodal driving or manipulation in complex conditions. However, these gripper-like actuators are usually made of rigid materials, and gear shaft systems are often needed to realize their multi-mode actuation. In recent years, soft continuum robots have been deeply explored, and their unique and attractive features have been demonstrated, such as a large degree of freedom and high biocompatibility [139,142]. Meanwhile, researchers have designed and fabricated various gripper-like soft actuators to realize various bionic movements, such as octopus bionic robot tentacles [143,144], trunk bionic robots [145], and bionic worm structures [146]. However, most of the previously constructed soft actuators are either pneumatically or hydraulically driven, which usually requires a large external control system with complex internal channels to achieve different actuation modes and prevent fluid leakage [147,148]. Currently, there is an urgent need to fabricate soft actuators using stimulus-responsive materials [149,150] that possess the potential to simplify the manufacturing and assembly process and reduce the complexity of control.

**Figure 5:**
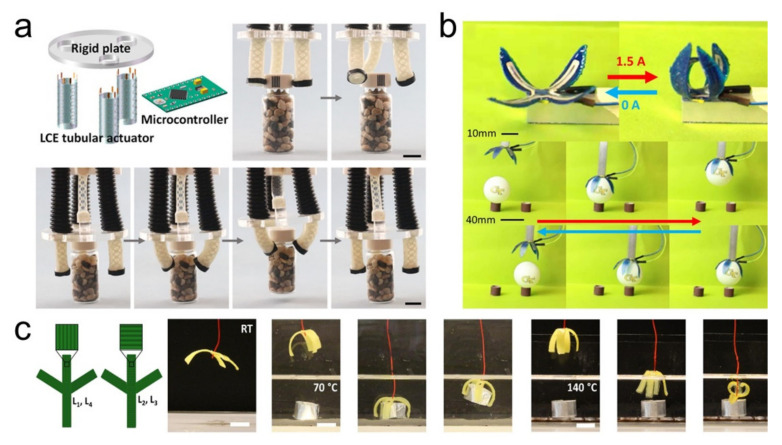
Grippers made by LCEs: (**a**) Multifunctional soft gripper with twisting and grasping functions [37] (Reproduced with permission from He, Q.; Wang, Z.; Wang, Y.; Minori, A.; Tolley, M.T.; Cai, S., Science Advances; Copyright 2019 American Association for the Advancement of Science.) (**b**) 4D printed four hinges soft robotic gripper picking and placing a ping pong ball [25] (Reproduced with permission from Roach, D.J.; Kuang, X.; Yuan, C.; Chen, K.; Qi, H.J., Smart Mater. Struct.; ©2018 IOP Publishing. Reproduced with permission. All rights reserved.) (**c**) A temperature-sensitive gripper. At a lower temperature (70 °C), the gripper’s lower-temperature response layer deforms to grab and lift the object; at a higher temperature (140 °C), the gripper’s higher-temperature response layer deforms and lowers the object [92] (Reproduced with permission from Davidson, E.C.; Kotikian, A.; Li, S.; Aizenberg, J.; Lewis, J.A., Adv. Mater.; Copyright 2020 John Wiley and Sons).

LCEs are good candidates for the preparation of gripper-like soft robots due to their large actuation stress and strain and versatile actuation modes. He et al. [37] reported an LCE-based tubular actuator with multiple actuation modes, controlled by externally applied electrical potential. By integrating three tubular actuators, LCE artificial muscle film, and a circular plate, a soft robot capable of grasping and releasing 50 mg vials was successfully developed. In order to show the application prospect of the flexible tubular actuator, they further made a cordless soft robot incorporated with vehicle power supply and microcontroller that can achieve various motions just driven by LCE tubular actuators. (Figure 5a). In another work [47], they realized grasping a bottle cap and lifting a cardboard by employing a soft robot that used fluid-driven disulfide-bonded LCE actuators as driving components. Roach et al. [25] developed a new type of LCE ink tha can be used for 3D printing directly at room temperature. The structures printed by this ink can achieve 48% reversible mechanical actuation. Therefore, this strategy allows for the integration of smart materials and 3D printing to create more complex shape changes and functional structures, including folding boxes, soft robot grippers, and sign language hands (Figure 5b). Saed et al. [92] developed a series of 4D printable nematic LC inks through a two-stage, one-pot thiol-ene “click” reaction. The physical properties of the printed LCEs can be controlled by varying the spacer, crosslinker, and mesogen concentration in the precursors, resulting in ultra-low actuation temperatures. In addition, the combination of multiple LCEs in a single printing structure will produce intelligent actuators with continuous shape changes. By integrating LCEs with different phase transition temperatures into the gripper, the intelligent response of the LCE gripper under different temperature conditions can be realized (Figure 5c). By mimicking flytrap, Wani et al. [151] demonstrated an autonomous light-driven graspable robot. This artificial flytrap is based on the reversible shape change of the photosensitive LCEs, which were fabricated onto the tip of an optical probe. Powered by light, this tiny fiber-tip device can grasp any micro-object with arbitrary shape. In addition, this artificial flytrap can act as an acquirer to accurately grasp the target object by distinguishing the different dynamics of the measured object.

### 3.3. LCEs for Moving Robots

In recent years, automated machines and smart robots have attracted more attention due to their intelligence and versatility. Typically, the actuators based on LCEs usually present a certain simple deformation in response to external stimulus, while the gripper-like robots can achieve some grasping actions similar to the human body. In addition, the LCE robots that can realize the typical move actions are also an important part of the smart robots. A summary of the actuation mechanism, structures, and functions of various soft robots is given in Table 2.

**Figure 6:**
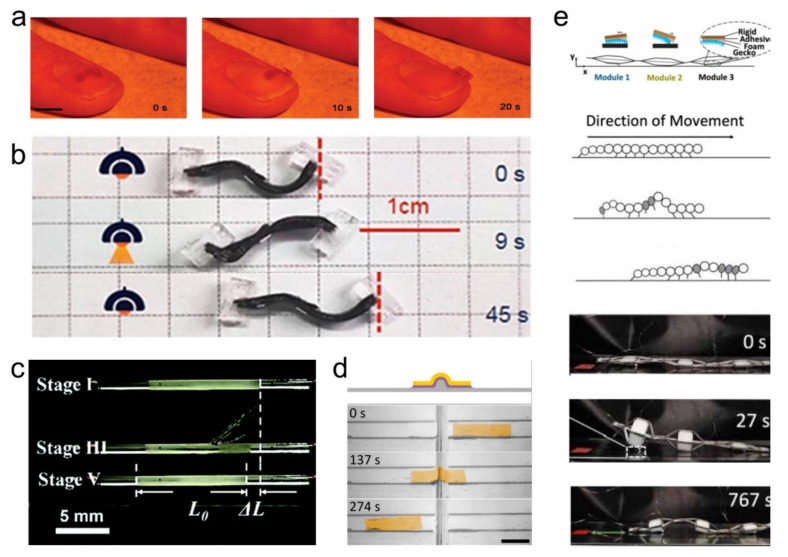
Worm-inspired LCE actuators: (**a**) Caterpillar robot motion on the nail of a human finger under the irradiation of 488 nm laser [85] (Reproduced with permission from Zeng, H.; Wani, O.M.; Wasylczyk, P.; Priimagi, Macromol. Rapid Commun.; Copyright 2018 John Wiley and Sons.), (**b**) The images of the serpentine robot moving under NIR light [156] (Reproduced from Ref. [156] with permission from The Royal Society of Chemistry.), (**c**) LCE capillary placed in a glass tube moving when tube heating [84] (Adapted from [84] with permission from The Royal Society of Chemistry.), (**d**) Snail-inspired LCE actuator climbing over an obstacle [29] (Reproduced with permission from Rogóż, M.; Dradrach, K.; Xuan, C.; Wasylczyk, P., Macromol. Rapid Commun.; Copyright 2019 John Wiley and Sons.), (**e**) Schematic of the LCE crawler made of three Sarrus, the robot crawling diagram modules, and representative experimental images of the caterpillar locomotion actuator [158] (Adapted with permission from Minori, A.F.; Fernandes, A.; He, Q.; Glick, P.; Adibnazari, I.; Stopol, A.; Cai, S.; Tolley, M.T., Smart Materials and Structures.; ©2020 IOP Publishing. Reproduced with permission. All rights reserved).

There are some strategies that can be used to control the motion of LCE soft robots. Global control by regulating the light stimulus is a promising strategy, during which a non-uniform force is usually required to drive the actuating unit. Typically, such uneven forces can be realized by moving on specially constructed surfaces (e.g., blazed grating) or manufacturing typical robots with unique structures. When the driving light is switched periodically, periodic shape changes will be generated, and the friction caused by the asymmetry of the robot structure will promote the crawling motion of the robot. Zeng et al. [85] prepared a millimeter-scale light-driven inching walker based on alignment-engineered LCE film and employing Disperse Red 1 as a photoresponsive filler. This miniature inching robot is composed of three alternately arranged sector segments, assembled into an “Ω”-shaped geometric structure. Therefore, driven by visible light, it can imitate the micromotion of the caterpillar to perform different movements on rough substrates (e.g., blazed grating, paper surface, and the nail of a human finger) (Figure 6a). Wang et al. [156] designed a novel snake-mimic actuator with bilayer LCE ribbon and two serrated feet. Under repeated on/off near-infrared stimulation, the actuator can move forward depending on the reversible shape deformation between the S-curve structure and the reverse S-curve structure, which is similar to snake motion (Figure 6b).

Controlling the local deformation of materials by external stimulations (heat, light, electricity) is another strategy for LCE robots to achieve much more precise motions. Liu et al. [84] demonstrated a series of LCE capillaries with biomimetic peristaltic function. By moving the heat source, LCE capillaries can perform peristaltic crawling locomotion in the glass tube (Figure 6c). Rogóż et al. [163] fabricated a natural scale light-driven walking robot that can achieve caterpillar-like movements. By controlling the position of the laser beam, a series of actions, such as climbing, squeezing through a slit, and pushing the micro-objects, can be realized. Inspired by snails, they further developed an optically driven LCE millimeter-scale crawler. Assisted by artificial mucus (glycerin, ethylene glycol, and microscope immersion oil), the LCE crawler can move smoothly on horizontal, vertical, and inverted surfaces and can climb over an obstacle under laser beam stimulation (Figure 4d) [29]. Minori et al. [158] introduced a self-folding system to drive complex folding motions through simple control of mono-domain LCEs (Figure 6e). This system is based on multiple linkage mechanisms and accompanied by independently controlled stretchable heaters. The crawling of the robot (Figure 6f) can be achieved by sequential controlling heaters to generate a wave of actuation along its length.

**Figure 7:**
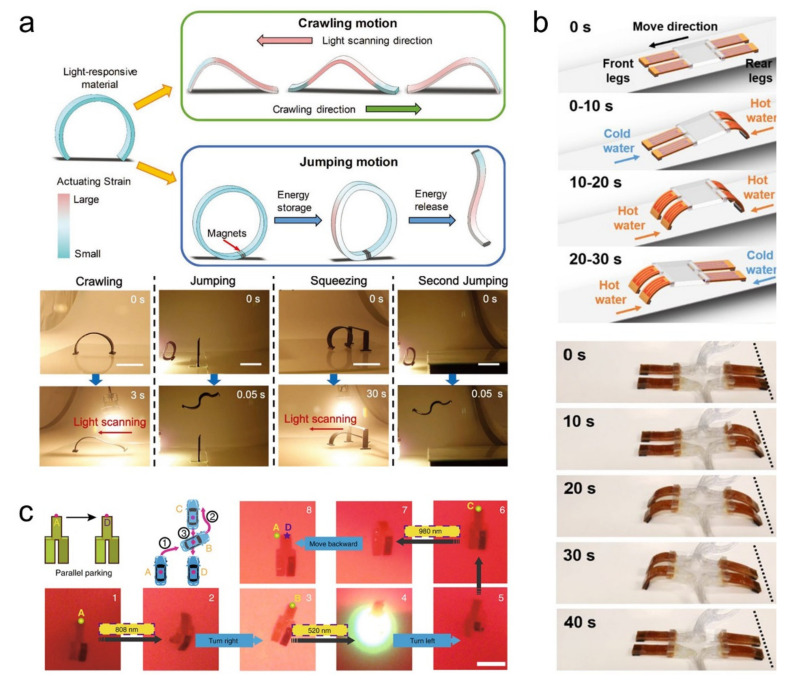
(**a**) Schematic illustration of the arch shape soft robot moving and crawling and jumping motion, and video frames of crawling, jumping, squeezing, and second jumping of the arch shape soft robot powered by light [36] (Reproduced with permission from Ahn, C.; Liang, X.; Cai, S., Adv. Mater. Technol.; Copyright 2019 John Wiley and Sons.), (**b**) Schematic illustration of the soft locomoting robot with automatic and programmable control, and images of the soft robot during moving [47] (Reprinted with permission from He, Q.; Wang, Z.; Wang, Y.; Song, Z.; Cai, S. Recyclable and Self-Repairable Fluid-Driven Liquid Crystal Elastomer Actuator. ACS Appl. Mater. Interfaces 2020, acsami.0c10021, doi:10.1021/acsami.0c10021. Copyright (2020) American Chemical Society.), (**c**) Schematic illustration and Photographs of the LCE walker robot parallel parking [155] (Reproduced with permission from Zuo, B.; Wang, M.; Lin, B.-P.; Yang, H.,Nat Commun; published by 2019 Springer Nature Limited).

Crawling robots struggle with complex action tasks. Therefore, some LCE robots with more advanced functions have been developed to handle more complex tasks. Ahn et al. [36] imitated the movement mode of fruit fly larva to design a soft robot that can realize multi-mode movement. They adopted a power amplification strategy called the spring-motor-latch system, which uses two small magnets at both ends of the active material to store energy and then release it at the right time to achieve greater instantaneous energy output (Figure 7a). The soft robot prepared by this strategy can realize multi-mode movement, including crawling, squeezing, and jumping powered by light (Figure 7a).

Soft robots with controllable motion have always been a hot research spot in the field of moving robots. He et al. [47] prepared fluid-driven disulfide LCE actuators through facile laminate manufacturing enabled by a dynamic bond exchange reaction. Benefitting from a heating/cooling mechanism, this robot can be operated in a wide temperature range and produce a large cyclic actuation at a frequency of 1 Hz. Based on this newly developed actuator, a soft robot system that can realize controllable and programmable motion is constructed (Figure 7b). Zuo et al. [155] reported a strategy for constructing a multi-stimulus responsive hierarchical LCE soft actuator system, which can not only perform controllable multi-directional moving but also possesses different shape deformation modes. In this study, three kinds of near-infrared dual-wavelength modulated actuators are demonstrated, including a bidirectional switch, a dual motion mode deformer, and bidirectional walker and a multi-directional walker robot. (Figure 7c). The rolling motion is one of the most common motions. Recently, the rolling motion has been implemented by LCE robots. Zuo et al. [162] designed an electro-driven soft actuator that can perform continuous rolling on a conductive track. This bilayer actuator composed of CB and LCE is driven by uneven stress caused by the heat generated by CB powder under the action of Joule-heating. The actuator uses the dynamic connection between the guide rail and the actuator to a certain extent to avoid the limitation caused by the wires of the traditional electrothermal actuator. Cheng et al. [164] created versatile 3D actuators and microrobots with photomechanical movements by light-responsive LCN sheets and the kirigami technique. The soft robot allows manipulation with light along a predesigned 2D trajectory, and it can climb a slope of up to 6°. The design principle described in these above papers opens up a new way to explore the new application of LCE soft actuator.

### 3.4. LCEs for Electric Devices

Flexible electronics have gained considerable interest in recent years due to their special characteristics and potential applications in wearable electronics and smart robots. Currently, the development of smart robots is driving the need to combine their key electronic components with intelligent responsive joints or motion modules to achieve multi functionality under more complex and harsh working conditions. LCEs, with unique characteristics of stimuli-responsive and reversible deformation, are super candidates for combining flexible electronics with intelligent response.

**Figure 8:**
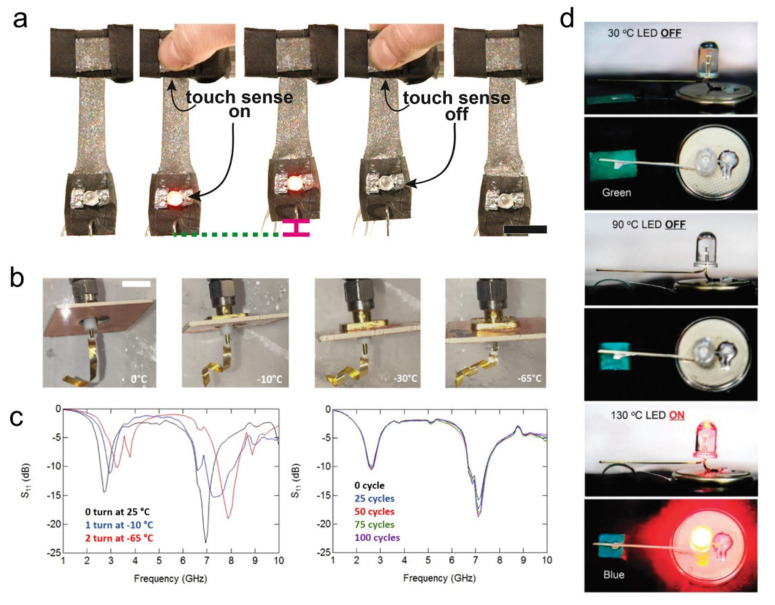
(**a**) LM–LCE composites function as a conductive wire to run current through an LED, as a transducer to sense touch, and as a Joule-heated actuator to lift a weight. An LED turns on when the sensing composite responds to touch, and internal Joule-heated actuation is activated [166] (Reproduced with permission from Ford, M.J.; Ambulo, C.P.; Kent, T.A.; Markvicka, E.J.; Pan, C.; Malen, J.; Ware, T.H.; Majidi, C. A, Proc Natl Acad Sci USA; published by 2019 National Academy of Sciences.) (**b**) Photographs of LCE composite antenna shape changes at different temperatures. (**c**) Measured reflection coefficient of the LCE antenna as temperature decreases(left); and measured frequency response of the reflection coefficient from 0 to 100 cycles(right) [39] (Adapted with permission from Kim, H.; Gibson, J.; Maeng, J.; Saed, M.O.; Pimentel, K.; Rihani, R.T.; Pancrazio, J.J.; Georgakopoulos, S.V.; Ware, T.H. Responsive, 3D Electronics Enabled by Liquid Crystal Elastomer Substrates. ACS Appl. Mater. Interfaces 2019, 11, 19506–19513, doi:10.1021/acsami.9b04189. Copyright (2019) American Chemical Society.) (**d**) Photographs of the Ag NPs/LCE nanocomposite actuator driving the electric circuit at different view angles and temperatures [38] (Adapted from Ref. [38] with permission from the Centre National de la Recherche Scientifique (CNRS) and The Royal Society of Chemistry).

Inspired by natural soft tissue, which can achieve rich functions through the hierarchical structure and sequence of the molecular level and the micro level [165], Ford et al. [166] introduced a soft and multifunctional composite material combining the sensing, mechanical robust electronic connection and active deformation. The multifunctionality of this film is accomplished by embedding the LCE with liquid metal (LM) microdroplets, which allow for enhancements in thermal and electrical properties and achieve their electrical or thermal responses. In addition, the Joule-heated actuators, transducers for touch sensing, and circuit wiring for surface-mounted electronic components are further demonstrated (Figure 8a). The combination of sensing and driving may have a revolutionary impact on the use of LCEs in soft material engineering.

By combining conventional electronic devices with responsive LCE substrates, Kim et al. [39] designed and manufactured a series of 3D, responsive electronics, including conducting traces, MIM capacitors, and frequency shifting antennas (Figure 8b–c). In this research, they explored the cold response of the LCE substrates and achieved 3D transformation of the resultant electronics not by heating but instead by cooling below the crosslinking temperature. The application of these 3D responsive devices in wearable or implantable electronic products and cold chain monitoring RFID sensors is very promising.

Colorimetric sensors that can change color when stimulated by external stimuli bring more intuitive information feedback to intelligent systems. Inspired by chameleons and cephalopods, Shi et al. [38] fabricated a simple bilayer colorimetric sensor by integrating a silver nanoparticle monolayer with a stimuli-responsive LCE layer. The results indicated that the AgNPs array embedded in the LCE layer could spontaneously induce the homeotropic orientation of the LC molecules, resulting in a reversible shape response associated with blueshift of structural color at vertical and hybrid alignment states of the bilayer films. Therefore, this facile bilayer-structural device can exhibit color change with thermally-driven actuation. Furthermore, a multi-level alert device was demonstrated by connecting the colorimetric sensor to the circuit. When the temperature rises from 30 °C to 130 °C, the alarm device not only shows the off–on switch state of the LED but also the significant color change. (Figure 8d).

## 4. Conclusions and Outlook

Liquid crystal elastomers (LCEs) are unique materials that combine the anisotropic behavior of liquid crystals and the rubber elasticity of lightly crosslinked polymers [167]. Compared to traditional shape memory materials, LCEs are appealing candidates for soft actuators due to their extraordinary mechanical properties, good flexibility, anisotropic behavior, and reversible shape responses [5,41]. With the rapid development of technology, research on liquid crystal elastomers has made significant progress in the past few decades. We reviewed this progress from two aspects:(1) The recent synthesis and processing technology of LCEs have been summarized, which is essential for further molecular design and device optimization; (2) The current research progress of LCEs in soft robots, actuators, sensors, and flexible electronics is comprehensively introduced, and the correlation between molecular arrangement and action property of LCEs is briefly discussed. Although the exploration of these actuator and sensor applications plays an important role in the design and manufacture of next-generation wearable and smart automatic devices, there is still a long way to make fully integrated LCE-based smart systems (such as smart robots). The main challenges in this area are described below:

For soft materials such as LCEs, there is often a trade-off relationship between the features of “high actuation stress” and “large actuation strain”, both of which are key factors determining their actuating performances [40,168]. In addition, most intrinsic LCEs are thermotropic, so it is difficult to realize their multi-stimulus responses and remote control, such as light-driven actuations. Currently, the most recent works are focusing on physically doping a variety of functional nano-fillers (photothermal, electrothermal nano-materials, etc.) into LCE matrices to address these above issues. However, due to the poor dispersibility of the functional fillers in the LCE matrix and the weak filler–matrix interaction, phase separation in the composites will inevitably occur and, subsequently, handicap the performance of the resulting actuators [169,170,171,172,173].

Secondly, there are still many doubts about the basic understanding of the relationship between molecular arrangement and action property of LCEs, and it is still a challenge to accurately control the deformation or motion of LCEs. Furthermore, the research on the processing technology of LCEs is still in the initial stage. Although some novel 3D or even 4D printing technologies have been developed to process LCEs into sophisticated architectures, these methods are aimed at customized equipment, and there is still no good solution for mass production.

Recent years have witnessed a growing interest in the employment of LCEs in building up smart devices. With the progress in new types of LCE materials, advanced 3D micro/nanofabrication technologies, novel action schemes, and sophisticated manipulation strategies, LCE-based actuators may achieve rapid advancements and find more cutting-edge applications in the near future.

## Figures and Tables

**Table 2:** Various LCE-based moving robots and their performance.

Stimulation and Mechanism	Structure Type	Functional Materials	Programming Method	Behavior	Performance	Ref.
Fluidthermal (Thermotropic)	Homogeneous entity	-	Mechanical stretching	Grasping; locomoting	Moving at ~48 mm in 320 s.	[47]
Direct heating (Thermotropic)			3D printing	Self-propelling (rotating)	Moving at ~17 cm in 95 s.	[152]
			Alignment cell	Crawling	Moving at 0.31 mm s^−^^1^ with heat source moving 2.32 mm s^−^^1^	[84]
Visible light (Isomerization)	Homogeneous entity	Custom azobenzene dye	Mechanical stretching; Photolithographic process	Swimming	Swimming speed of 2.6 μm s^−^^1^; rotation speed of 1° s^−^^1^.	[153]
	Homogeneous entity	Azobenzene dye	Alignment cell	Rotating	Rotating at speed of 30 rmp	[154]
UV light (Isomerization)	Bilayer	Azobenzene dye	Alignment cell	Light-driven plastic motor	-	[112]
	Bilayer	Azobenzene dye	Mechanical stretching	Crawling and rolling	Crawling at 3.4 mm s^−^^1^ Rolling at 19.4 mm s^−^^1^	[74]
Visible light (Photothermal)	Homogeneous entity	CNTs	Mechanical stretching	Crawling; jumping; squeezing	Crawl at the speed of 0.7 mm s^−^^1^.	[36]
		Dye (Disperse Red 1)	Alignment cell	Locomoting	Moving at 0.25 mm s^−^^1^ in blazed grating; 0.15 mm s^−^^1^ in the paper.	[85]
					Locomoting in vertical glass surface (0.08 mm s^−^^1^), upside-down horizontal glass surface (0.3 mm s^−^^1^), and crossing the obstacle (0.3 mm s^−^^1^).	[29]
Visible and NIR light (Photothermal)	Multilayer; Bilayer	Dye (Disperse Red 1, Dye 1002, YHD796)	Mechanical stretching	Walking	Photo-guided parallel parking.	[155]
NIR light (Photothermal)	Bilayer with two serrated feet	Dye (YHD796C)		Locomoting	Moving at ~10 mm in 80 s.	[156]
	Fiber	CNTs		Rolling	Protect a fragile cargo jump from falling from high places (50 cm); rolling with carrying a cargo (7.5 times heavier than the robot wight).	[157]
Electricity (Eectrothermal)	Multilayer	Copper heater	Mechanical stretching	Walking; twisting; grasping	Moving the rigid plate and the weight (10 g) on top forward 5 cm in 15 min.; grasping and lifting 50 g vial.	[37]
	Multilayer; multiple reversible self-folding module			Crawling	Moving at ~25 mm in 800 s (without frictional pads); moving at ~175 mm in 800 s (with frictional pads).	[158]
	Multilayer			Walking	Maximum movement speed of 1.27 cm min^−1^.	[159]
	Bilayer	Gold heater		Crawling	Crawling with an average speed of 1.91 mm min^−1^	[160]
	Multilayer	stretchable heating coils		Moving object	Moving a ball Lifting 200 g objects	[161]
	Homogeneous entity	CNTs		Swinging	-	[14]
	Bilayer	Carbon black		Rolling	Moving at 1.6 mm s^−1^ under 50 V DC power.	[162]

## Data Availability

Not applicable.
